# Optimizing acute stroke outcome prediction models: Comparison of generalized regression neural networks and logistic regressions

**DOI:** 10.1371/journal.pone.0267747

**Published:** 2022-05-11

**Authors:** Sheng Qu, Mingchao Zhou, Shengxiu Jiao, Zeyu Zhang, Kaiwen Xue, Jianjun Long, Fubing Zha, Yuan Chen, Jiehui Li, Qingqing Yang, Yulong Wang

**Affiliations:** 1 Department of Rehabilitation, Shenzhen Second People’s Hospital, The First Affiliated Hospital of Shenzhen University Health Science Centre, Shenzhen, China; 2 Department of Radiation Oncology, Shandong Cancer Hospital and Institute, Shandong First Medical University and Shandong Academy of Medical Sciences, Shandong, China; 3 School of Rehabilitation Sciences, The Shandong University of Traditional Chinese Medicine, Shandong, China; Jahangirnagar University, BANGLADESH

## Abstract

**Background:**

Generalized regression neural network (GRNN) and logistic regression (LR) are extensively used in the medical field; however, the better model for predicting stroke outcome has not been established. The primary goal of this study was to compare the accuracies of GRNN and LR models to identify the most optimal model for the prediction of acute stroke outcome, as well as explore useful biomarkers for predicting the prognosis of acute stroke patients.

**Method:**

In a single-center study, 216 (80% for the training set and 20% for the test set) acute stroke patients admitted to the Shenzhen Second People’s Hospital between December 2019 to June 2021 were retrospectively recruited. The functional outcomes of the patients were measured using Barthel Index (BI) on discharge. A training set was used to optimize the GRNN and LR models. The test set was utilized to validate and compare the performances of GRNN and LR in predicting acute stroke outcome based on the area under the receiver operating characteristic curve (AUROC), accuracy, sensitivity, and the Kappa value.

**Result:**

The LR analysis showed that age, the National Institute Health Stroke Scale score, BI index, hemoglobin, and albumin were independently associated with stroke outcome. After validating in test set using these variables, we found that the GRNN model showed a better performance based on AUROC (0.931 vs 0.702), sensitivity (0.933 vs 0.700), specificity (0.889 vs 0.722), accuracy (0.896 vs 0.729), and the Kappa value (0.775 vs 0.416) than the LR model.

**Conclusion:**

Overall, the GRNN model demonstrated superior performance to the LR model in predicting the prognosis of acute stroke patients. In addition to its advantage in not affected by implicit interactions and complex relationship in the data. Thus, we suggested that GRNN could be served as the optimal statistical model for acute stroke outcome prediction. Simultaneously, prospective validation based on more variables of the GRNN model for the prediction is required in future studies.

## Introduction

Stroke is among the leading causes of morbidity and mortality globally [1]. Up to 70% of patients have a persistent disability, and more than 40% have a severe disability, which places tremendous economic burdens on the families of patients and society [2]. To mitigate pressure on the healthcare system, assist medical professionals in making optimal clinical decisions [3], assist therapists in setting realistic therapeutic goals, improve the quality of life and life expectancy of acute stroke patients [4], as well as inform educated decisions on posthospital service needs for patients [5], accurate prognostic prediction is imperative. Besides, accurate outcome predictions would also assist policymakers and care providers in setting appropriate healthcare goals and optimizing the allocation of medical resources [6].

Studies on the clinical outcomes of patients have been at the forefront of healthcare research. Logistic regression (LR) and artificial neural networks (ANNs) have been used to evaluate prognosis for patients with stroke [7, 8]. The application of predictive models in clinical practice requires high standards: they must feature simple calculations and obvious structure and be tested with an independent dataset for simplicity, applicability, and reliability [9]. However, these models have advantages and disadvantages. Some are too complex for use in the clinical context, and others are susceptible to bias [10]. Consequently, establishing a reliable and feasible model for stroke prognosis prediction is an important challenge for rehabilitation physicians [6].

LR is commonly used for developing predictive models for dichotomous outcomes in medicine [11]. Its popularity may be attributed to the determination of the relationship between dependent variables and one or more nominal, ordered, interval, or ratio-level predictors and understanding the effects of predictors on outcome variables [8, 12]. However, it has several limitations: it is limited to linear relationships and it is prone to similarity and variance errors [9]. Therefore, the application of LR to the prediction of prognosis in patients with acute stroke has limitations [11].

ANN is a relatively advanced computational model used in bio-medicine and medical fields, and it is a mathematical model inspired by biological neural networks [13]. ANN simulates the problem-solving process of the human brain by applying knowledge gained from experience to build a system of "neurons" that can make new decisions, classifications, and predictions [9]. At present, four main neural network models are used in clinical practice: the back-propagation neural network (BPNN), generalized regression neural network (GRNN), fuzzy neural network, and probabilistic neural network [14]. The BPNN has been widely used [15] but suffers from some limitations, such as slow convergence speed, a habit of falling into a local optimal solution, and difficulties with setting an optimal configuration [16]. More efficient architecture through the GRNN has been proposed, with better approximation capabilities, non-linear fitting, easy parameter setting, and accelerated learning, as compared with the BPNN [17]. Based on these characteristics, the GRNN is suitable for solving problems of non-linear function and is used for data regression and classification [16].

Reports on the application of GRNNs to the prognostic prediction of diseases have been increasing. One study investigating antimalarial activities of selected therapies against *Plasmodium falciparum* found the prediction accuracy of a GRNN model for test sets to be 88.9% and that of a support vector machine to be 87.3%, which were both higher than that of a traditional LR model [18]. However, there are only a few studies on the value of a GRNN model in predicting acute stroke outcomes. Therefore, this study aimed to compare the prediction accuracy of acute stroke outcome using the GRNN and LR models and to identify the variables predictive of the prognosis of acute stroke patients from clinical data.

## Materials and methods

### Ethics statement

This was a single-center retrospective study that used the data of a previous study on the value of grip strength on the unaffected side in patients with stroke [19]. In our previous study, we not only explored related indicators of cardiopulmonary function in stroke patients, but also assessed Barthel index (BI) scores on discharge. In the context, this data was further utilized to construct LR and GRNN models. The medical records on admission and the functional outcome data on discharge from stroke patients at the Rehabilitation Medicine Department of Shenzhen Second People’s Hospital between December 2019 and June 2021 were collected. This study was approved by the Ethics Committee of Shenzhen Second People’s Hospital (Ethics No. KS20191119005) and registered in the Chinese Clinical Trial Registry (Registration No. ChiCTR1900028228).

### Study population

A total of 216 acute stroke patients who met the inclusion criteria were included in this study. The inclusion criteria were as follows: (a) age ≥18 years old; (b) onset within 90 days; (c) diagnosis of stroke based on history and physical examination and computed tomography (CT) findings; (d) ischemic (infarction of the central nervous system) or hemorrhagic (spontaneous, non-traumatic bleeding) stroke. The exclusion criteria were as follows: missing data, such as incomplete biochemical tests after admission, and loss to BI scores at discharge. All patients (or their family members) were provided complete written and oral information about all study procedures.

### Study variables, outcomes on discharge, and missing data

The variables were screened based on previously published articles and clinical experience [6, 11, 20]. Accordingly, 25 clinical variables were used in this study, and the data of the 216 patients were randomly divided into training (n = 168, 80%) and test (n = 48, 20%) sets using the Kennard-Stone algorithm. These included demographic data: age (years), gender, body mass index (BMI) (kg/m^2^), National Institute Health Stroke Scale (NIHSS), Baseline Barthel Index (BI), and etiology; medical history of coronary heart disease, hypertension, diabetes and atrial fibrillation; social history (drinking and smoking); physical examination findings (systolic blood pressure, diastolic blood pressure, pulse); biochemical indicators: D-dimer (μg/mL), total cholesterol (mmol/L), triglyceride (mmol/L), high-density lipoprotein cholesterol (mmol/L), low-density lipoprotein cholesterol (mmol/L), hemoglobin (Hb, g/L), serum Na+ (mmol/L), serum K+ (mmol/L), creatinine (μmol/L), and albumin (g/L).

The baseline function and functional outcomes of acute stroke patients were measured using the BI scale on admission and discharge (28–30 days of hospitalization) in this study. BI is widely used in clinical prognosis assessment, and has been called the most common ADL assessment scale for adult rehabilitation [21, 22]. According to previous reports [23, 24], patients with a BI ≥60 on discharge were allocated to the good outcome group, while patients with a BI <60 were allocated to the poor outcome group. The BI scores of the patients on admission and discharge were evaluated by the same professional physiotherapists.

Data cleaning and preprocessing were performed before analysis. Discarding and forcibly replacing large amounts of missing data are unreasonable. Continuous covariates with less than 1% of missing digital data could be imputed with the mean value, but covariates with more than 1% of missing data were excluded. Missing categorical covariates were recorded as negative.

### Development of the LR model

The LR model is used for classification based on certain predictors [12]. The dependent variable of LR is usually a binary variable, such as the outcomes (good or bad) of acute stroke patients [6]. For this model, the probability of a stroke outcome, given as, in relation to *n* prognosis-related covariates, *X*_*1*_, *X*_*2*_, *X*_*3*_, *⋯*, is given as follows:

$$logit(p_{i})=ln\left(\frac{p_{i}}{1-p_{i}}\right)=\beta_{0}+\beta_{1}X_{1}+\cdots +\beta_{i}X_{i}=\beta_{0}+\sum_{i=1}^{n}\beta_{i}X_{i}$$

Here, *β_i_* is the intercept; *β*_1_, *β*_2_, and *β_i_* are the regression coefficients for the predictive variables *X*_*1*_, *X*_*2*_ and *X_i_*. The regression coefficients indicate the contributions of the predictive variables to the results. The regression coefficients and their standard deviations are generally based on the least-squares fitting method. The LR model usually includes only important predictors, and variables are usually selected through some form of backward or forward stepwise logistic regression analyses [8]. The statistical significance of a variable was determined based on its coefficient, using p-values of <0.05. Then, the probability of an outcome was calculated using the logistic equation, and this was used to identify cases with poor prognoses in the test set.

### Development of the GRNN model

**Fig 1:**
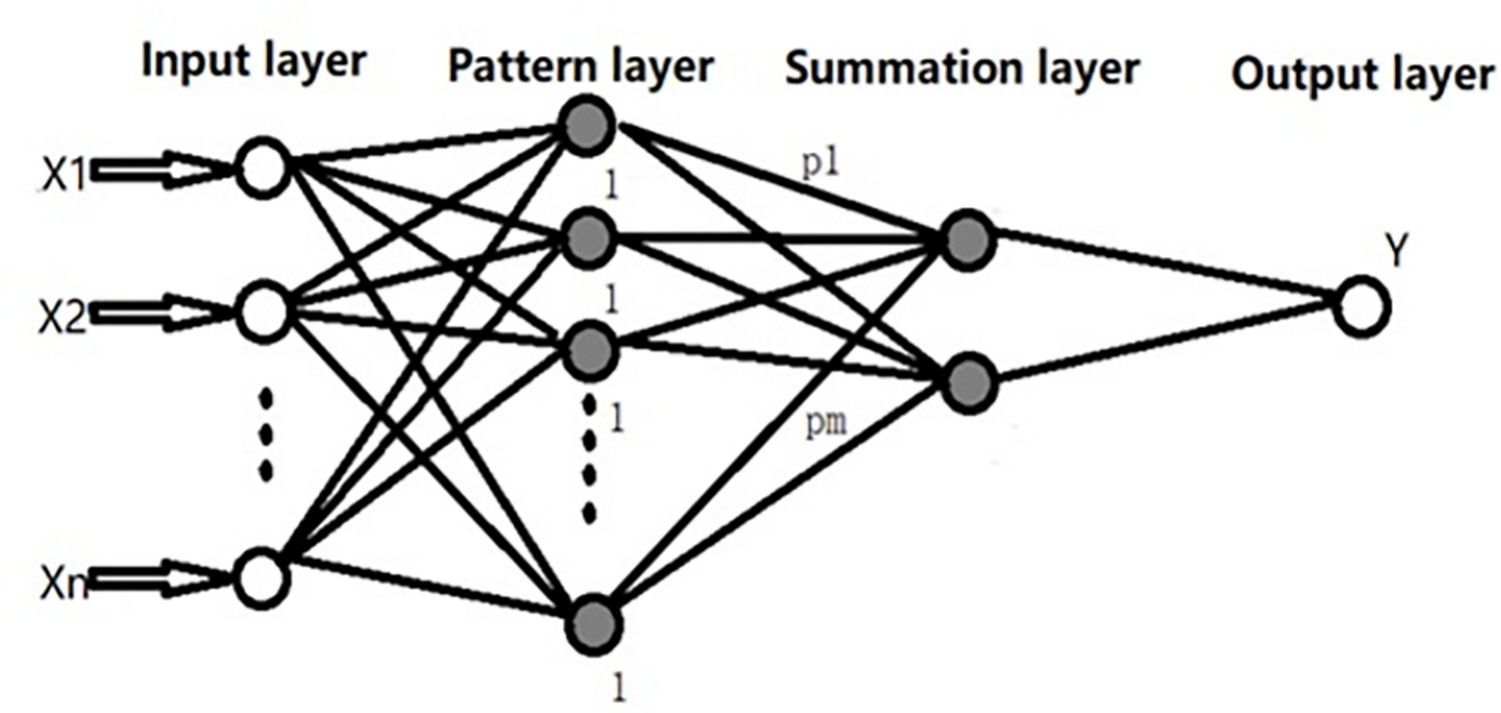
The structure of the GRNN.

The GRNN algorithm was first introduced by Specht [25] and considered to be used for classification problems where the target variable is continuous [26]. The algorithm does not reach the local minimum easily, and it has fewer adjustment parameters to be optimized. Therefore, it can be used to analyze large and unstable datasets [18]. The GRNN algorithm consists of four layers, as shown in Fig 1: input, pattern, summation, and output layers.

The variable definitions and interpretation are as follows:

$X={\left[x_{1},x_{2,},x_{3},\cdots ,x_{n}\right]}^{T}$: input vector of GRNN;

$Y={\left[y_{1},y_{2},y_{3},\cdots ,y_{k}\right]}^{T}$: output vector of GRNN;

*n*: the dimension of the vector variable *X*;

*k*: the dimension of vector variable *Y*;

In a GRNN model, the number of neurons in the input layer is equal to the dimension of the input vector [18].The input layer does not process the data but merely passes the input vector to the pattern layer. The number of neurons in the pattern layer is equal to that of the sample observations. Each neuron can be considered as a radial basis function and used to process a sample observation. The transfer function of the pattern layer, p_i_, serves as a Gaussian function as follows:

$$p_{i}=e^{-\frac{D_{i}{}^{2}}{2\sigma^{2}}}\;i=1,2,3,\cdots m$$

$$D_{i}{}^{2}={\left(X-X_{i}\right)}^{T}(X-X_{i})\;i=1,2,3,\cdots m$$

where *D*_i_ represents the distance between the point of prediction *X* and the training sample *X*_*i*_; *σ* is the width coefficient of the Gaussian function, a smoothness parameter; and *m* is the number of sample observations.

If the distance, *D*_i_, is large, $e^{-\frac{D_{i}{}^{2}}{2\sigma^{2}}}$ becomes small. Therefore, the contribution of the training samples to the prediction is relatively small. On the other hand, if the distance, *D*_i_, between the point of prediction *X* and the training sample *X*_*i*_ is small, $e^{-\frac{D_{i}{}^{2}}{2\sigma^{2}}}$ will become large. Therefore, the contribution of the training samples to the prediction is relatively large.

The summation layer uses two separate types of neurons for summation. The first type of calculation is:

$$S_{D}=\sum_{i=1}^{m}p_{i}$$

The second type of calculation is:

$$S_{E,j}=\sum_{i=1}^{m}Y_{i,j}p_{i}\;j=1,2,\cdots ,k$$

For the output layer, the number of neurons in the output layer is equal to the dimension of the output vector, and the output value of j is:

$$y_{j}=\frac{S_{E,j}}{S_{D}}\;j=1,2,\cdots k$$

### Statistics

The continuous and discrete variables are presented as mean ± standard deviation and median (interquartile range, IQR), respectively, and the categorical variables are presented as percentages. T-test of two independent samples and Mann-Whitney U test were used to compare the continuous and discrete variables, respectively. The categorical variables were analyzed using the chi-squared test. Significant variables from the univariate analysis were incorporated into the LR model, the non-significant variables were eliminated by backward selection, and the logistic regression model was finalized. The 95% confidence interval and the odds ratio were used to describe the relationships between variables. Hosmer–Lemeshow goodness of fit (χ^2^) was used to test the fit of the model.

A sensitivity analysis was used to evaluate the relative importance of input variables using the GRNN model and to rank the importance of variables. The global sensitivity of the input variables against the output variable was expressed as the ratio of the network error (sum of squares of residuals) with a given input omitted to the network error with the input included. The input variable with the highest global sensitivity was the variable with the greatest influence on the output variable.

The performance of the predictive model developed in our study was evaluated for discrimination ability using the test set; the discrimination ability was quantified using area under the curve (AUC) based on the actual BI scores of the acute stroke patients on discharge. Likewise, the accuracy, sensitivity, specificity, and the Kappa value were used to evaluate and compare the predictive performances of the LR and GRNN models.

We used SPSS version 26 (IBM, Armonk, NY, USA) to implement statistical analysis and construct the LR model. The GRNN model was generated using MATLAB 7.0. P<0.05 denoted statistical significance.

## Results

### Patient characteristics and univariate analysis

**Fig 2:**
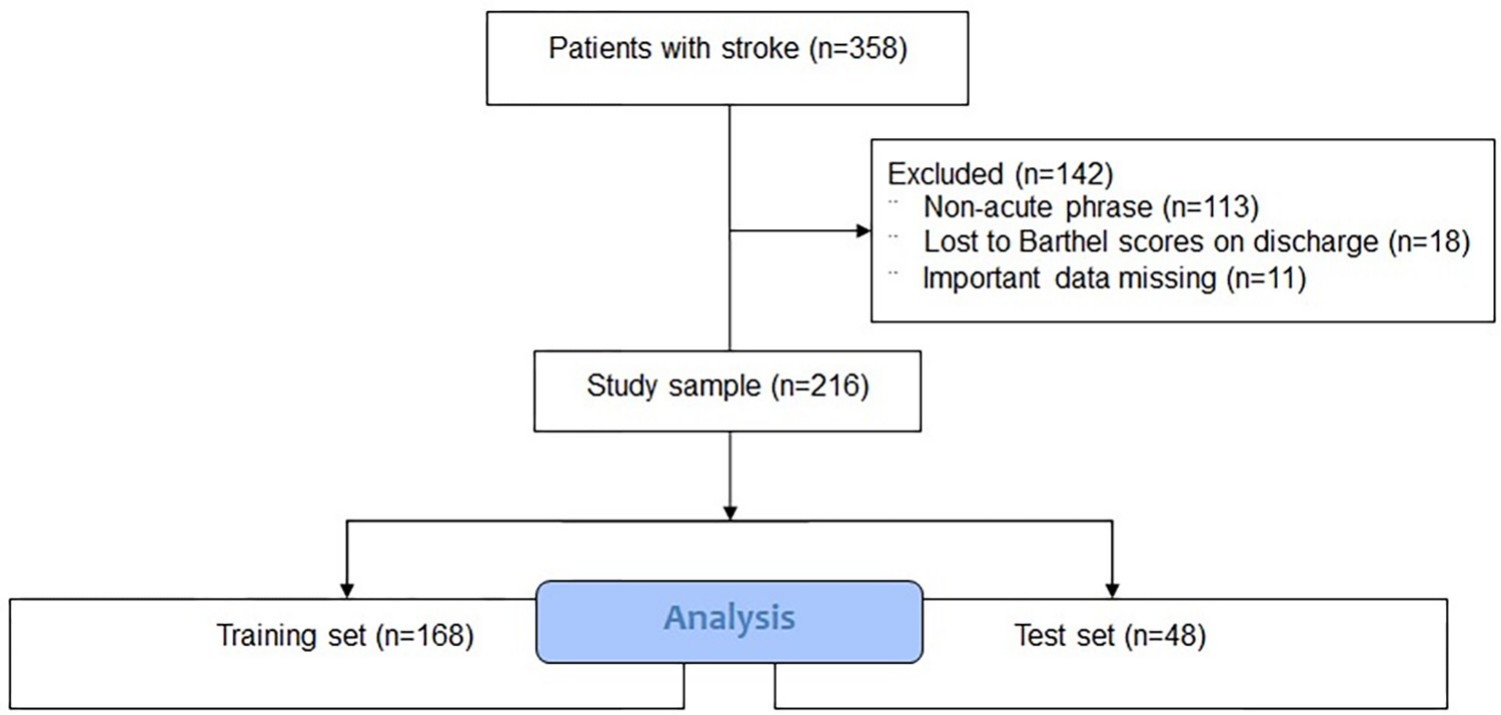
Study flowchart.

A total of 358 patients were screened; of these 11 patients were excluded due to missing data, 113 patients were excluded due to their non-acute stroke phase, and 18 patients were excluded due to lost the BI scores on discharge. In the end, 216 patients were included (168 patients in the training set and 48 patients in the test set) (Fig 2).

In the training set, 62.5% were for male patients with the mean age of 60.79±14.31 years; for the test set, 68.75% of the data were for male patients with a mean age of 58.44±14.90 years. (Table 1) In the training set, univariate analysis showed that gender, age, NIHSS, baseline BI, Hb, D-dimer, and albumin were significantly different in the good and poor outcome group of acute stroke patients (p<0.05). Compared with the patients with poor outcomes, the patients with good outcomes were much younger (58.01±14.40) and had greater BI scores on admission (58.97%), higher Hb (128.59±16.75) and albumin concentrations (39.83±3.74), and lower NIHSS (1.50 [0–5]) and D-dimer concentration (0.49 [0.31–0.87]) (Table 2).

**Table 1:** Distributions of characteristics at baseline among the training and test set.

	80% Training set	20% Test set
Variables	n = 168	n = 48
Age (years), mean ± SD	60.79±14.31	58.44±14.90
Male, n (%)	105 (62.50%)	33 (68.75%)
BMI (kg/m^2^), (IQR)	23.86 (21.29~25.38)	23.23 (21.39~26.22)
Current smoker, n (%)	57 (33.93%)	16 (33.33%)
Alcohol abuse, n (%)	60 (35.71%)	15 (31.25%)
Ischemic, n (%)	96 (57.14%)	20 (41.67%)
Hemorrhagic, n (%)	72 (42.86%)	28 (58.33%)
Baseline BI, Poor, n (%)	119 (70.83%)	38 (79.20%)
NIHSS, (IQR)	4 (0~11)	8 (2.00~20.75)
SBP (mmHg), mean ± SD	130.79±18.30	127.35±17.63
DBP (mmHg), (IQR)	80.00 (72.00~85.00)	80.00 (70.00~86.00)
Pulse, (IQR)	78.00 (72.00~85.75)	77.50 (70.00~87.50)
Coronary disease, n (%)	28 (16.67%)	13 (27.08%)
Hypertension, n (%)	136 (81.95%)	36 (75.00%)
Diabetes mellitus, n (%)	59 (35.12%)	10 (20.83%)
Atrial fibrillation, n (%)	13 (7.74%)	4 (8.33%)
D-dimer (μg/ml), (IQR)	0.71(0.39~1.40)	0.75 (0.39~2.74)
TC (mmol/L), (IQR)	3.42 (2.86~4.13)	3.50 (3.02~4.09)
TG (mmol/), (IQR)	1.24 (0.91~1.75)	1.13 (0.91~1.56)
HDL (mmol/L), (IQR)	0.97 (0.79~1.10)	1.05 (0.79~1.17)
LDL (mmol/L), (IQR)	1.99 (1.46~2.65)	1.91(1.59~2.50)
Hb(g/L), mean ± SD	122.82±19.66	122.93±19.01
Serum Na^+^ (mmol/L), (IQR)	139.40 (136.93~141.48)	140.85 (139.02~141.97)
Serum K+ (mmol/L), mean ± SD	4.00±0.40	3.89±0.44
Creatinine (μmol/L), (IQR)	58.75 (47.78~72.95)	62.30 (49.80~75.00)
Albumin (g/L), mean ± SD	39.05±4.41	39.50±5.69

Note: mean ± SD, mean ± standard deviation; IQR-interquartile range, BMI-body mass index, BI-Barthel, NIHSS-National Institute Health Stroke Scale, SBP-systolic blood pressure, DBP-diastolic blood pressure, TC-total cholesterol, TG-triglyceride, HDL-high density lipoprotein cholesterol, LDL-low density lipoprotein cholesterol, Hb-hemoglobin.

**Table 2:** Univariate analysis for the prognosis of acute stroke patients.

Variables	Good outcome (n = 78)	Poor outcome (n = 90)	P-value*
Age (years), mean ± SD	58.01±14.40	63.20±13.87	0.019*
Male, n (%)	60 (76.92%)	45 (50.00%)	0.000**
BMI (kg/m^2^), (IQR)	24.22 (21.6~25.42)	23.44 (20.97~25.39)	0.506
Current smoker, n (%)	25 (32.05%)	32 (35.56%)	0.632
Alcohol abuse, n (%)	33 (42.30%)	29 (32.22%)	0.097
Ischemic, n (%)	49 (47.44%)	47 (65.56%)	0.165
Hemorrhagic, n (%)	29 (15.38%)	43 (66.67%)	0.165
Baseline BI, Poor, n (%)	46 (58.97%)	3 (33.33%)	0.000**
NIHSS, (IQR)	1.50 (0~5)	8.00 (3~18)	0.000**
SBP (mmHg), mean ± SD	131.47±16.41	130.19±19.87	0.651
DBP (mmHg), (IQR)	80.00 (73.75~86.00)	78.50 (72.00~85.00)	0.273
Pulse, (IQR)	79.50 (72.00~86.50)	78.00 (70.75~85.00)	0.377
Coronary disease, n (%)	15 (19.23%)	13 (14.44%)	0.407
Hypertension, n (%)	62 (79.49%)	74 (82.22%)	0.653
Diabetes mellitus, n (%)	30 (38.46%)	29 (32.22%)	0.398
Atrial fibrillation, n (%)	5 (6.41%)	8 (8.89%)	0.547
D-dimer (μg/ml), (IQR)	0.49 (0.31~0.87)	0.95 (0.48~1.81)	0.000**
TC (mmol/L), (IQR)	3.30 (2.82~4.15)	3.53 (2.89~4.16)	0.287
TG (mmol/), (IQR)	1.20 (0.89~1.65)	1.29 (0.96~1.77)	0.416
HDL (mmol/L), (IQR)	1.00 (0.79~1.13)	0.97 (0.77~1.07)	0.298
LDL (mmol/L), (IQR)	1.87 (1.40~2.63)	2.05 (1.56~2.67)	0.477
Hb (g/L), mean ± SD	128.59±16.75	117.81±20.69	0.000**
Serum Na^+^ (mmol/L), (IQR)	139.95 (137.93~141.75)	139.20 (136.08~141.40)	0.151
Serum K+ (mmol/L), mean ± SD	3.96±0.39	4.05±0.41	0.178
Creatinine (μmol/L), (IQR)	62.95 (52.53~74.45)	57.00 (43.63~72.70)	0.052
Albumin (g/L), mean ± SD	39.83±3.74	38.36±4.82	0.031*

Note: mean ± SD, mean ± standard deviation; IQR-interquartile range, BMI-body mass index, BI-Barthel, NIHSS-National Institute Health Stroke Scale, SBP-systolic blood pressure, DBP-diastolic blood pressure, TC-total cholesterol, TG-triglyceride, HDL-high density lipoprotein cholesterol, LDL-low density lipoprotein cholesterol, Hb-hemoglobin.

*P<0.05

**P<0.01.

### Multiple logistic regression model

Multiple logistic regression was performed to analyze the independent associations among the indicators for the acute stroke outcomes. Based on the results of the univariate analysis, the dominant factors correlated with stroke outcomes, including age, NIHSS, Baseline BI, Hb, and albumin, were incorporated in the multiple logistic regression analysis. As shown in Table 3, age (OR = 1.039, 95% CI = [1.003, 1.076], p = 0.035), NIHSS (OR = 1.111, 95% CI = [1.038, 1.190], P = 0.002), Baseline BI (OR = 0.027, 95% CI = [0.007, 0.114], p = 0.000), Hb (OR = 0.972, 95% CI = [0.946, 0.997], p = 0.032), and albumin (OR = 1.154, 95% CI = [1.013, 1.314], p = 0.031) were independently associated with stroke outcome. The Hosmer-Lemeshow goodness of fit test result was 5.448 (P = 0.709) with 8 degrees of freedom. Furthermore, we derived the predictive equation using the training set with the following LR parameters (*Pi* is the probability of an acute stroke outcome):

Pi = 1+1/ (1 + e^–X^) = where X = −3.483 + 0.038 [age (years)] +0.106[NIHSS scores]– 3.061 [Baseline BI scores]– 0.029[Hemoglobin (g/L) + 0.143[Albumin (g/L)].

**Table 3:** Multiple logistic regression of outcomes (Barthel<60).

Characteristics	B	Standard error	Wald	OR	95% CI for odds		P value
					Lower	Upper	
Age (years)	0.038	0.018	4.462	1.039	1.003	1.076	0.035*
NIHSS	0.106	0.035	9.242	1.111	1.038	1.190	0.002**
Baseline BI	-3.061	0.730	24.36	0.027	0.007	0.114	0.000**
Hb (g/L)	-0.029	0.013	4.614	0.972	0.946	0.997	0.032*
Albumin (g/L)	0.143	0.066	4.673	1.154	1.013	1.314	0.031*
Constant	-3.483	2.883	1.460	0.031			

Note: NIHSS-National Institute Health Stroke Scale, Hb-hemoglobin, BI-Barthel, OR-the odds ratio.

Multiple logistic regression by backwards selection.

*P<0.05

**P<0.01.

### Comparison of the two models

**Fig 3:**
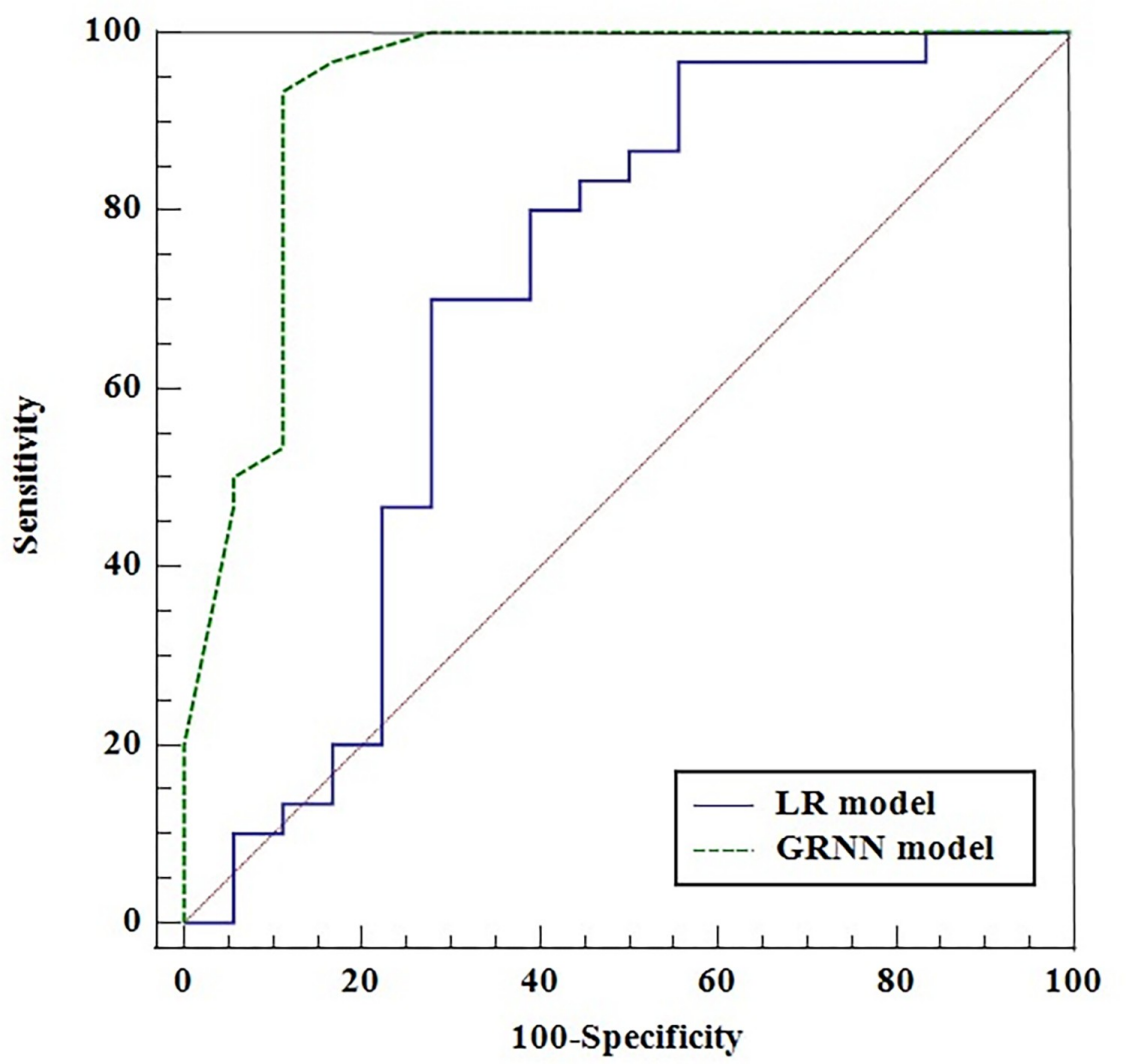
ROC curve of the LR and GRNN models for the test set.

The test set was used to compare the two models (Fig 3): the AUCs for the LR and GRNN models were 0.702 (95% CI: 0.553, 0.825) and 0.931 (95% CI: 0.820, 0.984), respectively. The LR and GRNN models had sensitivities of 0.700 (95% CI: 0.506, 0.853) and 0.933 (95% CI: 0.779, 0.992), specificities of 0.722 (95% CI: 0.465, 0.903) and 0.889 (95% CI: 0.653, 0.986), and accuracies of 0.729 and 0.896, respectively. Furthermore, the Kappa values for the LR and the GRNN models were 0.416 (95% CI: 0.149, 0.682) and 0.775 (95% CI: 0.589, 0.961), respectively (Table 4 and Table 5).

**Table 4:** Number of correct predictive values of LR and GRNN models.

			Predicted		
Model	Dataset	Observed	Good	Poor	Percentage
LR	Test set	Good	11	7	61.11%
		Poor	6	24	80.00%
		Total	17	31	
GRNN	Test set	Good	15	3	83.33%
		Poor	2	28	93.33%
		Total	17	31	

Note: LR- logistic regression, GRNN-generalized regression neural network.

**Table 5:** The performance of the LR and GRNN models.

	LR	GRNN
Input variables	Multiple variables	Multiple variables
AUCs	0.702 (0.553, 0.825)	0.931(0.820, 0.984)
Sensitivity	0.700 (0.506, 0.853)	0.933 (0.779, 0.992)
Specificity	0.722 (0.465, 0.903)	0.889 (0.653, 0.986)
Kappa value	0.416 (0.149, 0.682)	0.775 (0.589, 0.961)
Accuracy	0.729	0.896

Note: AUCs-area under the receiver-operating characteristic curves, LR-logistic regression, GRNN-generalized regression neural network.

### Global sensitivity analysis

The training data was used to calculate the variable sensitivity ratios (VSRs) for the GRNN model. The results indicated that the prognosis of patients with acute stroke is not determined by a single input variable but by a combination of multiple factors, among which baseline BI was the most influential or sensitive parameter affecting acute stroke outcome, followed by albumin and NIHSS (Table 6).

**Table 6:** Global sensitivity analysis of the GRNN model.

	Rank		
	First	Second	Third
Variable	Baseline BI	Albumin (g/L)	NIHSS
VSR	1.72	1.68	1.27

Note: variable sensitivity ratios-VSRs, BI-Barthel.

## Discussion

Stroke is a fatal and debilitating chronic disease associated with high mortality and morbidity rates and economic costs. Therefore, accurate, and reliable predictive models for prognostic predictions have clinical relevance. This is the first study to create a GRNN-based prediction model for acute stroke patients. GRNN model was a better model for predicting the short-term outcome of patients with acute stroke than the LR model. Secondly, we also found that Hb and albumin are important biochemical indicators of prognosis in patients with acute stroke by using an LR model.

Receiver operating characteristic curve analysis was used to compare the predictive performance of the LR and GRNN models, and a higher area under the receiver operating characteristic curve indicated a better predictive value. In our study, the GRNN model showed better performance based on the AUC (0.931vs 0.702), sensitivity (0.933 vs 0.700), specificity (0.880 vs 0.720), accuracy (0.896 vs 0.729), and Kappa value (0.775 vs 0.416) than the LR model. Therefore, we speculate that the GRNN model has better performance than the LR model for the prognostic prediction for acute stroke. Similar results have been reported by Liu et al [14], who reported that GRNN models may have better predictive ability than traditional multivariate LR models for dental caries based on the outcomes of an oral health epidemiology survey. Currently, GRNN models have shown use in predicting obstructive sleep apnea [27], quantitative structure–pharmacokinetic relationship properties of chemical agents [28], and so on. There are no previous studies that have constructed predictive GRNN models for patients with acute stroke. Yaru et al [11] compared the performances of BPNN models and LR models in predicting prognosis and found that the BPNN models had better performance indicated by higher AUC, accuracy, and specificity for acute ischemic stroke patients. The BPNN and GRNN are both types of ANN. A GRNN is a probabilistic neural network based on the knowledge generated by a Bayesian strategy for pattern classification, which does not need an iterative training procedure like a BPNN [29, 30]. GRNNs are reported to be superior to BPNNs in solving neutron spectrum problems [30]. Therefore, a GRNN was selected for the first time in this study to construct a prognostic model for acute stroke patients.

We considered that the GRNN model is superior to the LR model in predicting the prognosis of acute stroke patients for the following reasons. (1) GRNN is not affected by the interaction of factors and can include several dependent variables, which may have advantages in analyzing the complex variables involved in stroke [31]. (2) GRNN can "learn" without supervision, and those who have little experience in mathematical model construction can also build a model using software [7]. (3) GRNN is similar in principle to neurons of the human brain; it does not have difficulties related to the use of mathematics, and it is likely to make accurate predictions [32]. However, GRNN is considered a “black box”, which makes it difficult to determine its processes and how it arrives at a prediction or explain the disease-related variables in the predictive model [14]. LR, which is a commonly used medical statistical tool, also has its irreplaceable advantages, especially in the interpretability and simplicity of finding independent parameters associated with the prognosis of stroke patients [8, 33]. LR analysis is not the best at analyzing complex nonlinear relationships among variables, which has been proven in biology and complex epidemiological analysis [31].

In general, each model has its strengths and limitations, and the selection of a model should be based on its advantages and the intended purpose of the research. GRNN should be useful when the interest is in prediction, particularly when there are implicit interactions and complex relationships in the data, and it provides a better model fit than conventional logistic regression [7, 8]. For clinical interpretations, the LR model remains the better choice when statistical inferences must be drawn from the causal relationships between covariates and outcomes, and the estimated model regression coefficients provide odds ratios for easy identification of significant predictive indicators [34]. Thus, combining the best characteristics of LR and GRNN models may facilitate a more ideal predictive model for acute stroke patients.

Logistic regression analysis revealed that age, NIHSS, BI scores, Hb and albumin concentrations were independently associated with the prognosis of acute stroke. It is well-known that the prognosis of acute stroke is worse with increasing age due to reduced immunity and physiological function. Aged stroke patients may have higher mortality, longer hospital stay, and poorer quality of life than their younger counterparts [35]. Bielewicz et al. [6] reported that the BI scores assessment on day 10 was predictive of outcomes 3 months after ischemic stroke onset, indicating that the preexisting functional status was positively correlated with the prognosis of stroke patients. Besides, the NIHSS score has been shown to reflect neurological deficits and is considered valuable for predicting the outcome of stroke patients [6]. Based on global sensitivity analysis, BI scores and NIHSS scores influenced the prognosis of acute stroke patients, and so the evaluation results of these two scales should be paid attention to in the early stage.

Among the biochemical biomarkers, our study found that low albumin concentration was predictive of poor prognosis of acute stroke patients based on the univariate analysis; however, albumin was a risk factor based on the multivariate analysis, which may be related to the influence of the protective factors, BI and hemoglobin, on the predictiveness of albumin for the prognosis of acute stroke patients. Global sensitivity analysis indicated that albumin plays an important role in the prognosis of acute stroke. Hashem et al. [36], Aquilani et al. [37], and Xu et al. [38] reported that the high dose of albumin positively affects the prognosis of ischemic stroke patients, which were consistent with the results of this study. Albumin is often regarded as a marker of nutritional status and a negative phase protein that decreases in concentration during injury [39]. In animal models of ischemic stroke, a high concentration of albumin was found to have a significant neuroprotective effect on focal cerebral ischemia [40]. The neuroprotective effect of albumin is mainly related to scavenging free radicals, preventing lipid peroxidation, or binding metal ions and free fatty acids [41]. In other respects, high doses of albumin can reduce the risk of sarcopenia [42]. Therefore, we can conclude that early correction of hypoalbuminemia may help reduce the risk of poor outcomes of stroke.

Otherwise, a high concentration of Hb was a predictor of good outcome of acute stroke patients in our study. Only a few studies on the relationship between hemoglobin and the prognosis of acute stroke patients are available. As Bellwald et al. [43] demonstrated, a decrease in Hb had a linear relationship with larger infarct sizes in ischemic stroke patients, indicating a poorer prognosis. Any cause of the decrease in Hb during acute stroke had a negative impact on prognosis [43]. However, other studies showed that a high baseline concentration of Hb was associated with a poor prognosis of ischemic stroke [44]. High concentrations of hemoglobin increase blood viscosity, thereby increasing blood pressure and worsening cardiovascular function [45]. Thus, the effect of Hb on stroke outcomes is a controversial issue that needs to be further clarified in larger prospective studies.

Our study had several limitations. First, the patient’s follow-up after discharge were not evaluated, and predictors of long-term outcome of the acute strokes may be different from on discharged outcome. Second, the evaluation of model performance in this study was based on available hospital data at the time, and researchers should consider other data for validation before using them on a large scale. Third, this was a retrospective study, and selection bias was inevitable; besides, the sample was small, and this may have limited the generalization of the research results. Fourth, we only registered the stroke patients within 3 months of onset; Hence, it is unclear if this model would be as optimal for patients with subacute and chronic stages of the disease. Fifth, GRNN algorithm needs sophisticated software, and the complexity and unfamiliarity with the networks have been major drawbacks. With future advancements in computer technology and rapid training algorithms, this limitation of neural computing should be overcome. However, the results presented in our study are relevant, and they can guide and inspire further research on clinical stroke prognosis models. Furthermore, future research should be placed on developing prospective studies, collect more variables and complete the long-term follow-up data in subacute and chronic stroke population on a large scale, to validate the performance of the GRNN model.

## Conclusion

The study showed that acute stroke outcome can be readily predictable by using the GRNN model combining age, BI, NIHSS, hemoglobin, and albumin concentrations on admission and that this model could outperform LR model. The GRNN model is an accurate and meaningful tool, which can optimize existing models to facilitate more accurate predictions will improve the efficiency and accuracy of clinicians in predicting acute stroke outcomes at discharge and managing it. To make the GRNN model more representative, further researches should consider to design the prospective study collecting more variables of acute stroke patients on a large scale.

## Supporting information

S1 File The data supporting this work. (XLSX)

S2 File Protocol in English. (DOCX)

S3 File TREND statement checklist. (DOCX)

S4 File Protocol in Chinese. (DOCX)
