# Supplementary material for: Optimizing acute stroke outcome prediction models: Comparison of generalized regression neural networks and logistic regressions
Source: PLoS One. 2022 May 11;17(5):e0267747. doi: 10.1371/journal.pone.0267747 (PMC9094516; doi:10.1371/journal.pone.0267747)
Supplement: S2 File — (DOCX) [file pone.0267747.s002.docx]

**优化急性脑卒中预后预测模型:广义回归神经网络和Logistic回归的比较**

**研究目的**

本研究采用广义回归神经网络(Generalized regression neural network, GRNN)系统和Logistic回归分析(logistics regression, LR))预测急性脑卒中预后情况。我们用Barthel指数(BI)被用来评估患者出院时的功能。首先我们用训练集对GRNN和LR模型进行优化和构建。再基于测试集的受试者工作特征曲线(AUROC)下面积、准确性、灵敏度和kappa值，验证和比较GRNN和LR模型在预测急性卒中预后方面的性能。

**研究背景:**

脑卒中是全球发病和死亡的主要原因之一，其中高达70%的患者患有持续性残疾，超过40%的患者患有严重残疾，给患者家庭和社会带来了巨大的经济负担。 为减轻医疗保健系统的压力，协助医疗专业人员作出最佳临床决定，协助治疗师设定现实的治疗目标，提高急性中风患者的生活质量和预期寿命，以及为患者的出院后服务需求提供有效的指导，建立准确的预后预测模型非常重要。

现有的基于临床和生化指标构建的传统的统计学模型仍难以预测急性脑卒中的预后。因此，建立可靠可行的脑卒中预后预测模型是康复医师面临的重要挑战

LR是医学上开发二分结果预测模型的常用方法，它可以同时分析多个解释变量，减少混杂因素的影响。但是，它有几个局限性:响应变量的默认分布是正态分布; 它仅限于线性关系; 而且它容易产生相似性和方差误差。 因此，LR在预测急性脑卒中患者预后方面可能存在局限性。GRNN模型是一种新型的计算机模型，可以通过模仿生物神经系统，根据经验识别复杂数据。该模型具有分类能力强、近似能力强、学习速度快等优点。在其他临床医学中，如选择性治疗恶性疟原虫的抗疟活性方面，GRNN模型的分类模型有很好的准确性，并优于LR模型。因此，我们首先尝试构建GRNN模型，并与LR模型对急性脑卒中预后预测结果进行比较。

**研究设计**

研究类型:观察性实验

实际参与者:216受试者

观察模型:病例对照

时间类型:回顾性研究

研究题目：优化急性脑卒中预后预测模型:广义回归神经网络和Logistic回归的比较

研究开始日期: 2019年12月

实际初步完成日期: 2021年6月

实际研究完成日期: 2021年8月

**团体和人群**

**组织/组**

急性中风的结果，训练集

急性中风的结果，测试集

干预和治疗

利用训练集构建基于临床和生化指标的LR和GRNN模型。 然后，利用测试集根据受试者工作特征曲线下面积(AUROC)、准确性、灵敏度和kappa值来比较两个模型的性能。

**结果测量**

**主要结果测量:**

由同一专业理疗师评估出院时Barthel量表[时间框架:平均住院28~30天]。

**统计数据**

连续变量和离散变量分别用均数±标准差和中位数(四分位差，IQR)表示，分类变量用百分比表示。 连续变量和离散变量的比较分别采用两个独立样本的t检验和Mann-Whitney U检验。 采用卡方检验对分类变量进行分析.将单变量分析中的显著变量纳入LR模型，通过逆向选择剔除不显著变量，最终确定logistic回归模型。用95%置信区间和比值比来描述变量之间的关系。采用Hosmer-Lemeshow拟合优度(χ^2^)检验模型的拟合性。

利用测试集评价了本研究中建立的预测模型的辨别能力; 根据急性脑卒中患者出院时实际BI评分，使用曲线下面积 (area under the curve, AUC)对其识别能力进行量化。同样，准确性、敏感性、特异性和Kappa值被用来评估和比较LR和GRNN模型的预测性能。 我们使用SPSS version 26 (IBM, Armonk, NY, USA)来实现统计分析和构建LR模型。 利用MATLAB 7.0生成GRNN模型。 P<0.05为差异有统计学意义。

**合格标准**

**研究人群**

收集了2019年12月和2021年6月确诊为急性脑卒中患者。

**入选标准**

(a)年龄≥18岁;

(b) 90天内发病;

(c)根据病史、体格检查和计算机断层扫描(CT)发现诊断中风;

(d)缺血性(中枢神经系统梗塞)或出血性(自发性、非创伤性出血)中风。

**排除标准**

资料缺失，如入院后生化检查不完整，出院时BI评分缺失。

**研究地点**

**位置**

深圳大学附属第一医院、深圳市第二人民医院康复科，中国，518028。

**赞助商和合作者**

深圳市第二人民医院

**调查人员**

曲晟、周明超、焦胜修、张泽宇、薛凯文、龙建军、查甫兵、陈媛、李节惠、杨青青、王玉龙
