# Supplementary material for: Optimizing acute stroke outcome prediction models: Comparison of generalized regression neural networks and logistic regressions
Source: PLoS One. 2022 May 11;17(5):e0267747. doi: 10.1371/journal.pone.0267747 (PMC9094516; doi:10.1371/journal.pone.0267747)
Supplement: S4 File — (DOCX) [file pone.0267747.s004.docx]

**Optimizing acute** **stroke outcome prediction models: comparison of generalized regression neural networks and logistic regressions**

**Study Description**

Brief Summary:

This study was to predict the acute stroke outcome using generalized regression neural network (GRNN) system and logistic regression (LR) analysis. The functional outcome of the acute were measured using Barthel Index (BI) on discharge. A training set was used to optimize the GRNN and LR models. The test set was utilised to validate and compare the performances of the GRNN and LR models in predicting acute stroke outcome based on the area under the receiver operating characteristic curve (AUROC), accuracy, sensitivity, and the Kappa value.

Detailed Description:

Stroke is among the leading causes of morbidity and mortality globally. Up to 70% of patients have a persistent disability, and more than 40% have a severe disability, which places tremendous economic burdens on the families of patients and society. To mitigate pressure on the healthcare system, assist medical professionals in making optimal clinical decisions, assist therapists in setting realistic therapeutic goals, improve the quality of life and life expectancy of acute stroke patients, as well as inform educated decisions on posthospital service needs for patients, accurate prognostic prediction is imperative.

Existing model based on clinical and biochemical indicators still was difficult to predict the acute stroke outcome. Thus, establishing a reliable and feasible model for stroke prognosis prediction is an important challenge for rehabilitation physicians

LR is commonly used for developing predictive models for dichotomous outcomes in medicine, which can simultaneously analyze multiple explanatory variables and reduce the influence of confounding factors. However, it has several limitations: the default distribution of response variables is normal; it is limited to linear relationships; and it is prone to similarity and variance errors. Therefore, the application of LR to the prediction of prognosis of acute stroke patients has limitations. The GRNN model is a novel computer model, can identify patterns in complex data based on experience by imitating the biological nervous system. This model has several advantages over other models, including classification ability, approximation ability, and learning speed. In the other clinical medicine, as if antimalarial activities of selected therapies against Plasmodium falciparum, the GRNN model predictive accuracy was satisfying enough and outperformed than LR model. So, we firstly tried to construct the GRNN model, and compare with the LR model for prediction of the acute stroke outcome.

**Study Design**

Study Type : Observational

Actual Enrollment : 216 participants

Observational Model : Case-Control

Time Perspective : Retrospective

Official Title : Optimizing acute stroke outcome prediction models: comparison of generalized regression neural networks and logistic regressions

Study Start Date : December, 2019

Actual Primary Completion Date : June, 2021

Actual Study Completion Date : August, 2021

**Groups and Cohorts**

**Groups/Cohorts**

the acute stroke outcome, training set

the acute stroke outcome, test set

**Intervention/Treatment**

Using training set to construct the LR and GRNN models based on the clinical and biochemical indicators. Afterwards, the test set was used to compare the two models performance based on the area under the receiver operating characteristic curve (AUROC), accuracy, sensitivity, and the Kappa value.

**Outcome Measures**

**Primary outcome measures:**

Barthel scores on discharge [ Time Frame: average 28~30 days of hospitalization] were evaluated by the same professional physiotherapists.

**Statistics**

The continuous and discrete variables are presented as mean ± standard deviation and median (interquartile range, IQR), respectively, and the categorical variables are presented as percentages. T-test of two independent samples and Mann-Whitney U test were used to compare the continuous and discrete variables, respectively. The categorical variables were analyzed using the chi-squared test. Significant variables from the univariate analysis were incorporated into the LR model, the non-significant variables were eliminated by backward selection, and the logistic regression model was finalized. The 95% confidence interval and the odds ratio were used to describe the relationships between variables. Hosmer–Lemeshow goodness of fit (χ^2^) was used to test the fit of the model.

The performance of the predictive model developed in our study was evaluated for discrimination ability using the test set; the discrimination ability was quantified using area under the curve (AUC) based on the actual BI scores of the acute stroke patients on discharge. Likewise, the accuracy, sensitivity, specificity, and the Kappa value were used to evaluate and compare the predictive performances of the LR and GRNN models. We used SPSS version 26 (IBM, Armonk, NY, USA) to implement statistical analysis and construct the LR model. The GRNN model was generated using MATLAB 7.0. P<0.05 denoted statistical significance.

**Eligibility Criteria**

**Study Population**

Diagnosed the acute stroke patients from December 2019 and June 2021 were collected.

**Inclusion Criteria**

(a) age ≥ 18 years old;

(b) onset within 90 days;

(c) diagnosis of stroke based on history and physical examination and computed tomography (CT) findings;

(d) ischemic (infarction of the central nervous system) or hemorrhagic (spontaneous, non-traumatic bleeding) stroke.

**Exclusion Criteria**

Missing data, such as incomplete biochemical tests after admission, and loss to BI scores on discharge.

**Contacts and Locations**

**Locations**

Department of Rehabilitation, Shenzhen Second People's Hospital, The First Affiliated Hospital of Shenzhen University, Shenzhen, China, 518028.

**Sponsors and Collaborators**

Shenzhen Second People's Hospital

**Investigators**

Sheng Qu and Mingchao Zhou, Shengxiu Jiao, Zeyu Zhang, Kaiwen Xue, Jianjun Long, Fubing Zha, Yuan Chen, Jiehui Li, Qingqing Yang, Yulong Wang
